# Genome-wide identification of the heat shock transcription factor gene family in two kiwifruit species

**DOI:** 10.3389/fpls.2023.1075013

**Published:** 2023-09-20

**Authors:** Jing Tu, Muhammad Abid, Juan Luo, Yi Zhang, Endian Yang, Xinxia Cai, Puxin Gao, Hongwen Huang, Zupeng Wang

**Affiliations:** ^1^ College of Life Science, Nanchang University, Nanchang, China; ^2^ Lushan Botanical Garden, Chinese Academy of Sciences, Jiujiang, China; ^3^ Wuhan Botanical Garden, Chinese Academy of Sciences (CAS), Wuhan, China

**Keywords:** high temperature, *Hsf* gene family, *A. chinensis*, *A. eriantha*, gene expression profiles, heat stress

## Abstract

High temperatures have a significant impact on plant growth and metabolism. In recent years, the fruit industry has faced a serious threat due to high-temperature stress on fruit plants caused by global warming. In the present study, we explored the molecular regulatory mechanisms that contribute to high-temperature tolerance in kiwifruit. A total of 36 *Hsf* genes were identified in the *A. chinensis* (Ac) genome, while 41 *Hsf* genes were found in the *A. eriantha* (Ae) genome. Phylogenetic analysis revealed the clustering of kiwifruit *Hsfs* into three distinct groups (groups A, B, and C). Synteny analysis indicated that the expansion of the *Hsf* gene family in the Ac and Ae genomes was primarily driven by whole genome duplication (WGD). Analysis of the gene expression profiles revealed a close relationship between the expression levels of *Hsf* genes and various plant tissues and stress treatments throughout fruit ripening. Subcellular localization analysis demonstrated that GFP-AcHsfA2a/AcHsfA7b and AcHsfA2a/AcHsfA7b -GFP were localized in the nucleus, while GFP-AcHsfA2a was also observed in the cytoplasm of *Arabidopsis* protoplasts. The results of real-time quantitative polymerase chain reaction (RT-qPCR) and dual-luciferase reporter assay revealed that the majority of *Hsf* genes, especially *AcHsfA2a*, were expressed under high-temperature conditions. In conclusion, our findings establish a theoretical foundation for analyzing the potential role of *Hsfs* in high-temperature stress tolerance in kiwifruit. This study also offers valuable information to aid plant breeders in the development of heat-stress-resistant plant materials.

## 1 Introduction

The life span of plants is significantly influenced by various abiotic stresses, with temperature playing a critical role in affecting plant growth and development (Imran et al., 2021). The issue of temperature-associated damage has been heightened due to global warming, posing a significant threat to agricultural production and product quality on a global scale (Srivastav et al., 2021). In response, plants have evolved various mechanisms to mitigate the detrimental effects of heat stresses (Hasanuzzaman et al., 2013; Zhao et al., 2020). Among these mechanisms, *Hsfs* (heat shock transcription factors) serve as the central regulator of high-temperature stress in plants (Ohama et al., 2017; Wang et al., 2023). Beyond responding to temperature, *Hsfs* have been shown to regulate plant responses to other types of abiotic stress and play crucial roles in plant development (Begum et al., 2013; Xin et al., 2017; Zang et al., 2019; Khan et al., 2023). Prior studies have demonstrated that members of the *Hsf* family stimulate their transcription under high temperatures by binding to heat shock-responsive elements of downstream genes, thereby enhancing the plant’s resistance to heat (Ikeda et al., 2011; Yoshida et al., 2011).

Hsf proteins are typically comprised of six distinct, conserved domains: the DNA binding domain (DBD), nuclear localization signal (NLS), oligomerization domain (OD), nuclear export signal (NES), C-terminal activator peptide motif (AHA), and repressor domain (RD) (Wang et al., 2023). Nonetheless, variations are observed in these conserved domain architectures across different Hsf protein subgroups (Scharf et al., 2012; Guo et al., 2016). *Hsf* family members are divided into three primary groups (Nover et al., 2001). Group A Hsf proteins are further categorized into nine subgroups (A1-A9), and members of these subgroups typically harbor five conserved domains, including DBD, OD, NLS, NES, and AHA (Nover et al., 2001; Scharf et al., 2012; Guo et al., 2016). Group B Hsf proteins, which are divided into five subgroups (B1-B5) (Liao et al., 2022), lack AHA and NES domains in their sequences (Nover et al., 2001; Scharf et al., 2012; Guo et al., 2016). Group C, which usually includes two subdivisions (C1-C2), consists of the DBD, OD, and NLS domains (Nover et al., 2001; Scharf et al., 2012; Guo et al., 2016). The sequence variations and domain architecture determine the functional differences of Hsf proteins (Nover et al., 2001; Scharf et al., 2012; Guo et al., 2016). Group A is the most substantial in terms of Hsf proteins, with its gene functions thoroughly documented (Andrasi et al., 2021). Members of group A typically elevate plant tolerance to high temperatures by increasing the expression of downstream *HSP* genes (Jacob et al., 2017). Interestingly, HsfB1 acts simultaneously as a co-activator of HsfA1a in several HSPs and as a transcriptional repressor in other *Hsfs*, such as HsfA1b and HsfA2 (Bharti et al., 2004; Ikeda et al., 2011; Fragkostefanakis et al., 2019).

The genus *Actinidia*, more commonly recognized as kiwifruit, consists of 54 species and 75 taxa (Huang, 2016). Although the cultivation of most kiwifruit cultivars originates from *A. chinensis* (Ac), *A. eriantha* (Ae) has recently been utilized for the development of new kiwifruit cultivars (Huang and Ferguson, 2007). Notably, the cultivars originating from Ae contain an extraordinarily high vitamin C content compared to those developed from Ac (Drummond, 2013). Moreover, Ae exhibits a higher level of tolerance to elevated temperatures than Ac (Zhong et al., 2018).

Members of the *Hsf* gene family have been identified in various plant species, such as *Arabidopsis thaliana*, *Solanum tuberosum* L., *Solanum lycopersicum* L., and *Oryza sativa* L. (Nover et al., 2001; Wang et al., 2009; Tang et al., 2016; Yang et al., 2016). However, until now, there have been no reports concerning the identification and structural characterization of *Hsf* gene family members within the kiwifruit species. Here, we systematically identified and characterized the *Hsf* gene family members in both kiwifruit species. Additionally, we further investigated the expression bias of the *Hsf* gene family in different tissues and stages of plant development, and inferred their potential roles in regulating kiwifruit responses to ethylene treatment. Our findings provide foundational information regarding the structure characters and potential function of *Hsf* genes within kiwifruit, acting as a valuable resource for researchers aiming to develop kiwifruit cultivars with enhanced tolerance to heat stress.

## 2 Materials and methods

### 2.1 Genome-wide identification of *Hsf* genes in kiwifruit

The genomic, coding, and protein sequences for *AcHsfs* and *AeHsfs* were obtained from the Kiwifruit Genome Database (http://kiwifruitgenome.org/) (Yue et al., 2020). The protein sequences for AtHsfs were collected from The *Arabidopsis* Information Resources Database (TAIR, https://www.arabidopsis.org/) (Lamesch et al., 2012), while the protein sequences for OsHsfs and SlyHsfs were retrieved from the Heat Stress Transcription Factors (HEATSTER, http://www.cibiv.at/services/hsf/) database (Berz et al., 2019). To identify homologous sequences in both kiwifruit genomes (Ac and Ae), the protein sequences of AtHsfs, OsHsfs, and SlyHsfs were used as queries in the BLASTp search. Blast hits with a score of ≥ 100 and an e-value of ≤ 1×e^-10^ were considered candidate Hsf proteins from the Ac and Ae genomes. The candidate genes from AcHsfs and AeHsfs were then identified using the HMMER 3.0 (https://www.ebi.ac.uk/Tools/hmmer/) based on the Hidden Markov Model (HMM) of the HSF_DNA-binding protein (PF00447) (Potter et al., 2018). To determine the conserved domains in kiwifruit *Hsfs*, the Conserved Domain Database (CDD, https://www.ncbi.nlm.nih.gov/Structure/cdd/cdd.shtml), Pfam and Simple Modular Architecture Research Tool (SMART, http://smart.embl.de/) were employed (Lu et al., 2019; Letunic et al., 2021; Mistry et al., 2021). The motifs and domains were analyzed based on the HEATSTER database (Berz et al., 2019). The protein sequences containing the HSF_DNA-binding protein were subjected to subsequent analyses.

### 2.2 Physical properties of Hsf proteins in kiwifruit

The length, molecular weight, grand average of hydropathicity (GRAVY), and theoretical isoelectric point (pI) of the kiwifruit Hsf proteins were calculated using the ExPASy server (http://web.expasy.org/protparam/) online tool (Duvaud et al., 2021).

### 2.3 Structure analysis of kiwifruit *Hsf* genes

The distribution pattern of intron and exon in kiwifruit *Hsf* genes was analyzed using the Gene Structure Display Server (GSDS 2.0, https://gsds.cbi.pku.edu.cn/), based on the coding and genomic sequences. In addition, the MEME tool (https://meme-suit.org/tools/meme/) was employed to identify a maximum of 12 conserved motifs in AcHsfs and AeHsfs (Bailey et al., 2009).

### 2.4 Phylogenetic analysis of kiwifruit *Hsfs*

Multiple sequence alignments were conducted for AtHsf, OsHsf, SlyHsf, AcHsf, and AeHsf proteins using Clustal X with default parameters (Larkin et al., 2007). The phylogenetic tree was constructed using the neighbor-joining (NJ) method with 1,000 bootstrap replicates through the MEGA X software (Kumar et al., 2018).

### 2.5 Chromosome location, duplication, and synteny analysis of kiwifruit *Hsf* genes

Information about the length of a chromosome and the location of genes on the chromosomes was extracted from a GFF file of kiwifruit genomes using an in-house Perl script (Morris et al., 2008). To visualize the genes on chromosomes, the MG2C v2 (Map Gene2chromosome) (http://mg2c.iask.in/mg2c_v2.0) online software was utilized. The identification of duplication events in kiwifruit *Hsfs* was performed using the MCScan X program with default parameters (Wang et al., 2012). The TBtools software was employed to calculate the Ka (synonymous) and Ks (non-synonymous) substitution rates in duplicated gene pairs of kiwifruit *Hsfs* (Chen et al., 2020). The syntenic blocks within and between the Ae and Ac genomes were generated using the MCScan X software with default parameters (Wang et al., 2012), and the duplicated gene pairs of kiwifruit *Hsfs* were visualized using TBtools (Chen et al., 2020).

### 2.6 Expression profile of kiwifruit *Hsfs*

Four transcriptome datasets (PRJNA691387, PRJCA003268, PRJNA277383, and PRJNA514344) were retrieved from the NCBI (https://www.ncbi.nlm.nih.gov/) and NGDC (https://ngdc.cncb.ac.cn/). These datasets comprised samples from various plant tissues, different developmental stages of fruit, fruit treated with ethylene, and fruit treated with low temperature (**Table S5**). The kiwifruit genome ‘Ac’ was used as a reference for the reanalysis of the transcriptome datasets (Pilkington et al., 2018). The clean reads were aligned using HISAT2 (v2.0.1) (Kim et al., 2019), and STRINGTIE (v2.1.5) was used to assemble and quantify the reads (Pertea et al., 2015).

### 2.7 Plant materials and application of treatments

The fresh leaf samples of Ae ‘Maohua 1’ and Ac ‘Donghong’ were collected from Kiwifruit Plant Resource Nursery at Lushan Botanical Garden, Chinese Academy of Sciences, Nanchang, China. A portion of the samples was used for gene cloning, while the remaining samples were utilized for callus induction. The calluses were subjected to light and dark conditions for further investigations. *In-vitro* kiwifruit seedlings were grown and exposed to temperature intervals of 25°C, 28°C, 32°C, 37°C, and 42°C for 3 h in a growth chamber with 55% relative humidity. Each treatment was replicated thrice to achieve uniform results. Tobacco and *A. thaliana* plants were cultivated under growth conditions including a 16/8 h light/dark photoperiod, a temperature of 26°C, and a humidity level of 55%.

### 2.8 Total RNA extraction, cDNA synthesis, and RT-qPCR analysis

The Hipure Plant RNA Mini Kit (Magen, Shanghai, China) was utilized to extract total RNA from the samples, and the EasyScript One-Step gDNA Removal and cDNA Synthesis SuperMix (Transgen, Beijing, China) was employed to synthesize cDNA by following the manufacturer’s protocol. The reaction mixture for RT-qPCR was prepared according to the instructions of the MonAmpTM ChemoHS qPCR mix kit as follows: each 20 µl reaction mixture contained 1 µl of template cDNA, 1 µl of the qPCR mix (MonAmpTM ChemoHS), and 0.5 µl of each primer. The reaction conditions comprised an initial denaturation at 95°C for 5 min, followed by 45 cycles of denaturation at 95°C for 10 sec, annealing at 60°C for 20 sec, and extension at 72°C for 20 sec. The kiwifruit actin gene was used as a reference for data normalization. The relative expression was calculated by the 2^-△△Ct^ method (Livak and Schmittgen, 2001). The kiwifruit *AcActin* gene (Acc08081) and *AeActin* gene (DTZ79_07g10460) were selected as the reference genes (Huang et al., 2020; Abid et al., 2022). All the primer pairs used for RT-qPCR can be found in **Table S4**.

### 2.9 Cloning and subcellular localization analysis of *AcHsfs*

The coding sequences (CDS) of AcHsfA2a and AcHsfA7b were cloned by using specific pairs of primers AcA2a/AcA7b-EGFP F/R and EGFP-AcA2a/AcA7b F/R from cDNA template of Ac ‘Donghong’ kiwifruit (**Table S4**). The amplification was performed using the following thermal cycling conditions: initial denaturation at 98°C for 30 s, followed by 35 cycles of denaturation at 98°C for 10 s, annealing at 57°C for 5 s, and extension at 72°C for 10 s. The resulting CDS, without stop codon, were inserted into an empty pGreen vector to generate AcHsf-eGFP and eGFP-AcHsf constructs. An empty pGreen vector was used as a positive control. The constructed plasmids were then introduced into the *A. tumefaciens* strain EHA105. Finally, the bacterial culture was introduced into tobaccos and *A. thaliana* plants (with nuclear localization marker 35s-nls-linker-mkate) for performing sub-cellular localization (Yoo et al., 2007; Zhao et al., 2017). The transformed leaves and protoplasts were visualized under a laser scanning confocal microscope Olympus IX83 (Olympus, Tokyo, Japan).

### 2.10 Transient over-expression and luciferase assay

To study the activity of the candidate gene promoter, it was cloned into the pGreenII-0800-LUC vector. The resulting recombinant product was then transformed into the *A. tumefaciens* strain EHA105. A blank pGreenII-0800-LUC vector was considered as a control. The bacterial culture was injected into the tobacco leaves by using a needleless syringe. The transformed plants were kept in the dark for 24 h and then transferred to normal light conditions for an additional 36 h. Finally, the plants were treated with different temperature intervals: 25°C, 37°C, and 42°C, each for 3 h. The LUC (Firefly luciferase) and REN (Renilla luciferase) values were measured using the Dual-Luciferase Reporter Assay System kit (Promega, Madison, WI, USA).

### 2.11 Statistical analysis

All statistical analyses were carried out on GraphPad Prism 9 software. The significance levels of data were checked by performing one-way ANOVA with the Dunnett posttest. Mean differences between groups were then examined using Tukey’s test, and mean differences were considered significant at *p ≤* 0.05.

## 3 Results

### 3.1 Identification of *Hsfs* family members in kiwifruit

We identified a total of 36 and 41 putative *Hsf* family members from the genomes of Ac (hereafter referred to as *AcHsf*) and Ae (hereafter referred to as *AeHsf*), respectively (**Figure S1** and **Table S1**). Notably, the kiwifruit species exhibited a higher abundance of *Hsf* genes compared to most reported plants (**Table S3**). Our findings demonstrated that all identified putative Hsf proteins in Ac and Ae possessed the conserved HSF_DNA-binding domain (**Figure S1**). Interestingly, we observed that several Hsf proteins in Ac and Ae also harbored other conserved domains (**Figure S1**), indicating their potential roles in defining the functions of specific genes. The CDS length of *AcHsfs* ranged from 177 bp (*AcHsfA4a*) to 2190 bp (*AcHsfB5a*), while that of *AeHsfs* ranged from 621 bp (*AeHsfA4d*) to 1536 bp (*AeHsfA1c*) (**Table S1**). In terms of protein length, AcHsf proteins varied from 59 to 730 amino acids (aa), and AeHsf proteins ranged from 207 to 512 aa (**Table S1**). Moreover, the predicted molecular weight of AcHsf proteins ranged from 6772.54 to 83845.65 kDa, and for AeHsf proteins, it ranged from 24202.95 to 56935.26 kDa (**Table S1**). Theoretical pI calculations revealed that the pI values of AcHsf proteins varied from 4.56 to 9.03, while for AeHsf proteins, the range was 4.64 to 8.96 (**Table S1**). Subcellular localization prediction indicated that both AcHsf and AeHsf proteins localize in the nucleus of plant cells (**Table S1**).

### 3.2 Chromosomal localization of kiwifruit *Hsfs*

**Figure 1:**
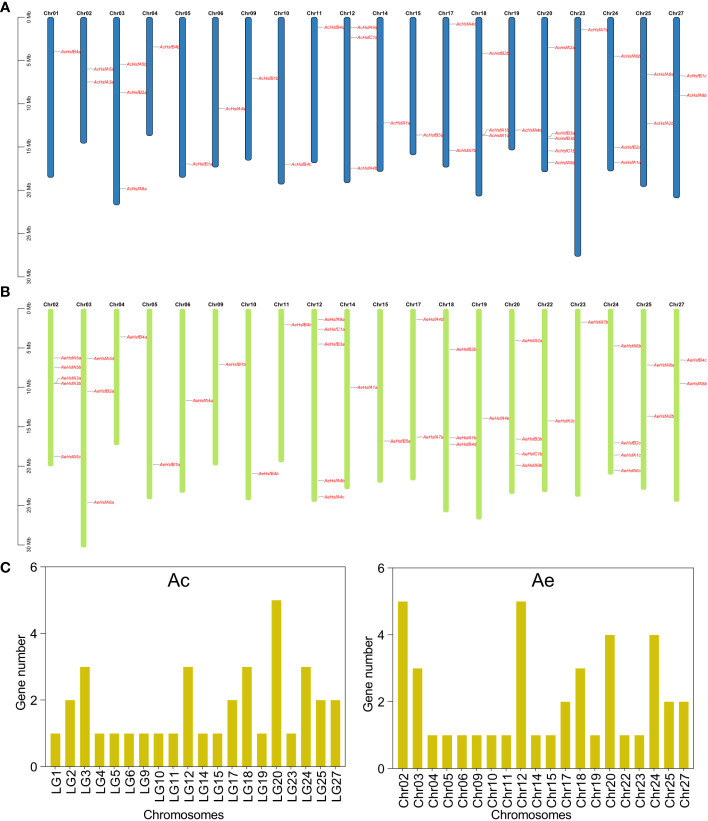
Chromosomal location of kiwifruit *Hsf* genes. **(A)** Location of *AcHsfs* on chromosomes, **(B)** Location of *AeHsfs* on chromosomes, and **(C)** Number of genes on each chromosome.

The 36 *AcHsf* genes were distributed randomly across 20 chromosomes in the Ac genome (**Figure 1A**). Our results revealed that chromosome 20 included the greatest number of *Hsf* genes, with five genes present. This was followed by chromosomes 3, 12, 18, and 24, which contained three genes each. Chromosomes 2, 17, 25, and 27 harbored two genes each, while the remaining chromosomes contained only one *AcHsf* gene (**Figures 1A, C**). Similarly, the 41 *AeHsf* genes exhibited an uneven distribution across 20 chromosomes in the Ae genome. Chromosomes 02 and 12 contained the highest number of *AeHsf* genes, with five genes each. Chromosomes 20 and 24 carried four *AeHsf* genes, while chromosomes 03 and 18 contained three *AeHsf* genes. Chromosomes 17, 25, and 27 contained two *AeHsf* genes, and the remaining chromosomes had a single *AeHsf* gene (**Figures 1B, C**).

### 3.3 Phylogenetic analysis of kiwifruit *Hsf* gene family

**Figure 2:**
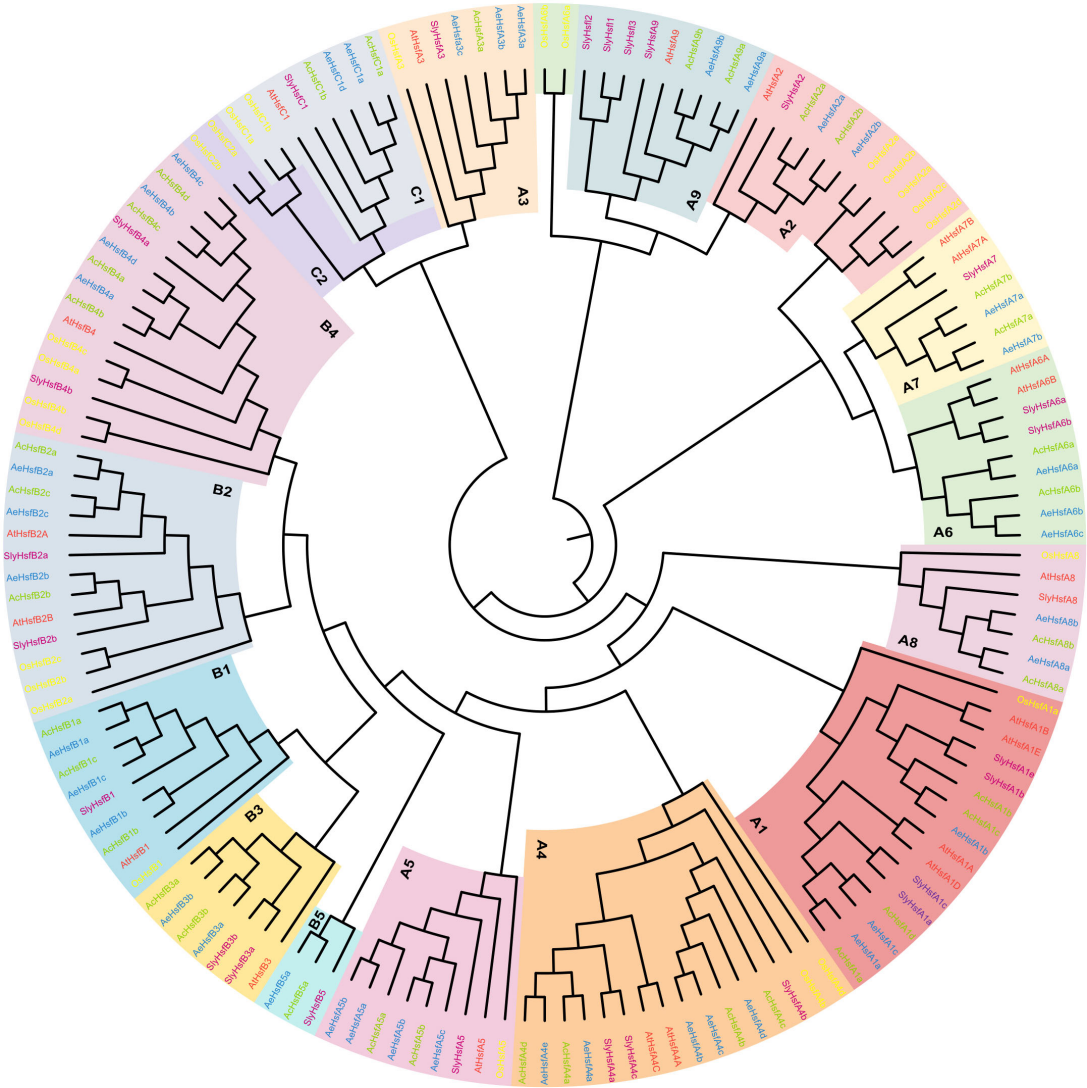
Phylogenetic analysis of Hsf proteins. Blue, green, yellow, purple, and red colors denote AeHsfs, AcHsfs, OsHsfs, SlyHsfs, and AtHsfs, respectively. Each subgroup of Groups A, B, and C was highlighted with different colors.

To explore the phylogenetic relationships and evolutionary patterns of *Hsf* genes in kiwifruit, we constructed a phylogenetic tree using the neighbor-joining (NJ) method. The tree included a total of 150 Hsf proteins, consisting of 36 AcHsfs from Ac, 41 AeHsfs from Ae, 25 OsHsfs from *Oryza sativa*, 27 SlyHsfs from *Solanum lycopersicum*, and 21 AtHsfs from *Arabidopsis*. Based on homology with AtHsfs, OsHsfs, and SlyHsfs, the kiwifruit *Hsfs* were classified into three main groups, namely groups A, B, and C (**Figure 2**). Group A was further subdivided into nine subgroups (A1-A9), while group B was divided into five subgroups (B1-B5) (**Figure 2**). Among the Hsfs from kiwifruit, 21 out of 36 *AcHsfs* and 26 out of 41 *AeHsfs* belonged to group A (**Figures 2**, **S2**). The subgroups within group A exhibited varying numbers of kiwifruit *Hsf* genes, ranging from four to eight. Subgroup A4 had the most kiwifruit *Hsf* genes, with four *AcHsfs* and five *AeHsfs* (**Figures 2**, **S2**). In group B, subgroup B4 contained the highest number of kiwifruit *Hsf* genes, with four *AcHsfs* and four *AeHsfs* (**Figures 2**, **S2**). Lastly, subgroup C1 consisted of two *AcHsfs* and two *AeHsfs genes* (**Figures 2**, **S2**).

### 3.4 Gene structure and conserved motifs analyses of kiwifruit *Hsfs*

**Figure 3:**
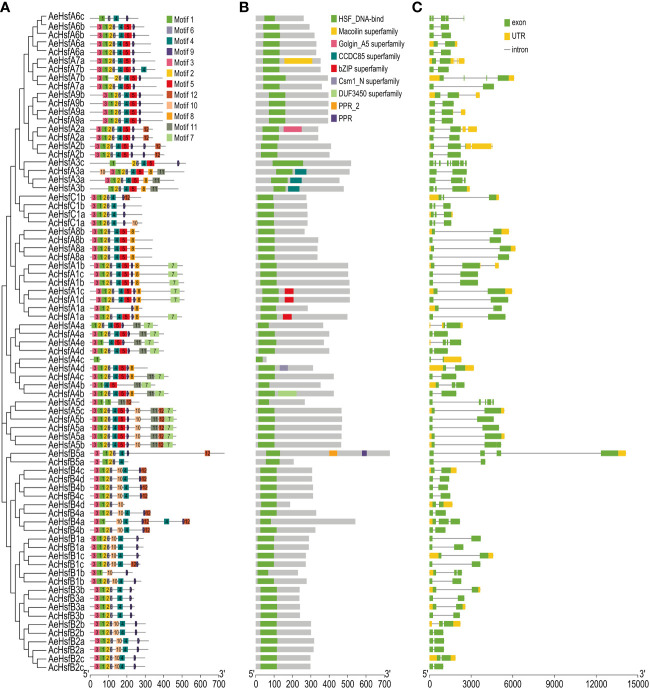
Structure analysis of kiwifruit Hsf genes. **(A)** Conserved motifs, **(B)** Conserved domains, and **(C)** Exon-intron distribution in genes.

The conserved domains and motif architectures of kiwifruit *Hsfs* were predicted by Pfam and MEME, respectively (**Figures 3A, B**). A total of 12 conserved motifs, labeled as motif 1 to motif 12, were identified for the kiwifruit Hsfs (**Figure 3A**). The number of motifs within group A varied from 1 to 11, within group B it ranged from 4 to 9, and within group C it varied from 6 to 7 (**Figure 3A**). The identification of certain motifs in kiwifruit Hsfs suggested the presence of a specific occurrence pattern. For instance, all *Hsf* genes in both kiwifruit species contained motif 1, while motifs 2, 3, 4, 6, and 9 were found in a majority of the *Hsf* genes (**Figure 3A**). We further confirmed that motifs 1, 2, 3, and 6 constitute a conserved HSF_DNA-binding domain by using the Pfam and CDD databases (**Figure S4**) (Lu et al., 2019; Mistry et al., 2021). Interestingly, motif 5 was exclusive to group A Hsfs, while motif 10 was particularly present in group B, except for subgroup B3 (**Figure 3A**). This implies that the presence or absence of specific motifs may play a role in the functional diversification of kiwifruit *Hsf* genes within particular groups.

The exon-intron structure of genes is an essential feature that provides insights into their evolutionary history, functional diversification, and classification (Zhu et al., 2009). The *AeHsf* genes possessed a higher number of exons compared to the *AcHsf* genes (**Figures 3C**, **S3A**). The exon numbers for *AcHsf* and *AeHsf* genes varied from two to eight, with *AeHsfA3c* from subgroup A3 having the highest number of exons (eight exons) (**Figures 3C**, **S3B**). This indicates that there are distinct gene structures between *AcHsfs* and *AeHsfs* (**Figure 3C**). The number of introns in a gene can also play a role in regulating gene function through alternative splicing of transcripts (Shang et al., 2017; Laloum et al., 2018). Most *AcHsf* genes (34 out of 36 *AcHsfs*) had only one intron, whereas *AeHsfs* exhibited a wide range of intron numbers, ranging from 1 to 7, suggesting the functional diversification observed in *AeHsf* genes may be influenced by alternative splicing events (**Figure 3C**).

### 3.5 Synteny and gene duplication analyses of kiwifruit *Hsfs*

**Figure 4:**
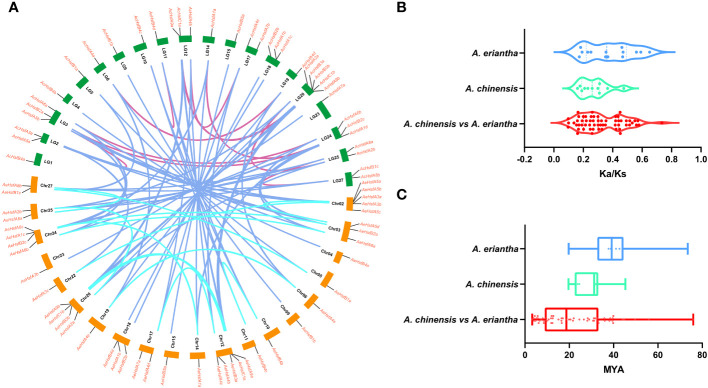
Collinearity analysis of kiwifruit Hsfs. **(A)** Circos plot for duplicated gene pairs. The chromosomes in Ac and Ae genomes are indicated in green and orange bars, respectively. Different colored lines indicate the duplicated gene pairs within and between both kiwifruit genomes, **(B)** Selection pressure experienced by duplicated gene pairs, and **(C)** Divergence time (T).

The addition or deletion of genes is the primary evolutionary source causing the expansion or contraction of a gene family (Magadum et al., 2013). There were 11 duplicated gene pairs in the Ac genome and 14 duplicated gene pairs in the Ae genome (**Figure 4A** and **Table S2**). The fact that all duplicated gene pairs experienced whole-genome duplication (WGD) events suggests that WGD accounted for the expansion of the kiwifruit *Hsf* gene family (**Table S2**) (Zwaenepoel and Van De Peer, 2019).

We estimated the selection pressure experienced by duplicated gene pairs as follows: Ka/Ks < 1 indicates purifying selection, Ka/Ks > 1 denotes positive selection, and Ka/Ks = 1 stands for neutral selection (Hurst, 2002; Zhang et al., 2006). In the present study, the selection pressure ranged from 0.1 to 0.5 for *AcHsfs* and 0.1 to 0.7 for *AeHsfs* (**Figure 4B** and **Table S2**). Our results suggested that duplicated gene pairs in kiwifruit had experienced purifying selection. The divergence time for *Hsf* paralog gene pairs in Ac and Ae ranged from 19.61 to 45.31 and 19.72 to 73.45 MYA (million years ago), respectively (**Figure 4C**). Additionally, the divergence time for ortholog gene pairs between the Ac and Ae genomes ranged from 3.26 to 75.93 MYA (**Figure 4C**). Notably, the divergence time for *Hsf* ortholog gene pairs between the Ac and Ae genomes was higher than that of *Hsf* paralog gene pairs in Ac (**Figure 4C**).

### 3.6 Expression patterns of kiwifruit *AcHsfs* in different tissues

**Figure 5:**
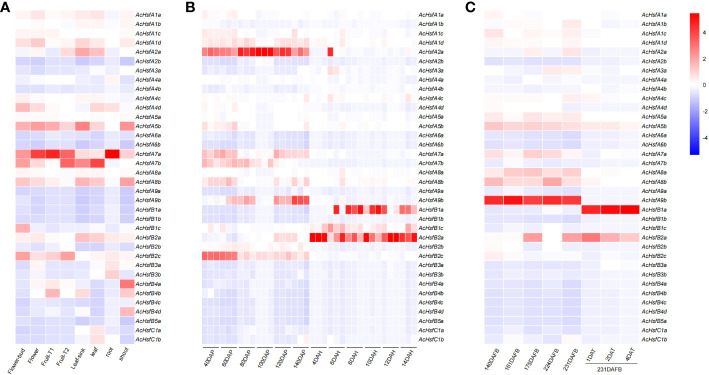
Expression profiles of kiwifruit *Hsfs* based on transcriptome data. **(A)** Expression profiles of *AcHsfs* in eight different plant tissues (fruit T1: no ethylene production, fruit T2: autocatalytic ethylene production), **(B)** Expression profiles of *AcHsfs* in different developmental stages of fruit (DAP, day after pollination; DAH, days after harvest), **(C)** Expression profiles of *AcHsfs* in different developmental stages of fruit and samples treated with ethylene (DAFB, days after the full bloom of fruit; DAT, day after being treated with ethylene).

We utilized four transcriptome datasets to evaluate the expression patterns of *AcHsfs*. The expression bias of *AcHsfs* was estimated across eight different plant tissues (flower buds, flowers, fruit T1: no ethylene production, fruit T2: autocatalytic ethylene production, leaf sink, leaves, roots, and shoots) (**Figure 5A**). Our analysis revealed highly tissue-specific expression profiles for kiwifruit *Hsfs*, including *AcHsfA1d*, *AcHsfA5b*, *AcHsfA7a*, *AcHsfA7b*, *AcHsfA8b*, *AcHsfB2a*, and *AcHsfB2c*. Specifically, the expression levels of *AcHsfA7a* were relatively higher in flowers, fruits T1, and roots compared to other genes tested (**Figure 5A**). Furthermore, we investigated the expression of *AcHsfs* at different fruit developmental stages and found that several genes, namely *AcHsfA1d*, *AcHsfA2a*, *AcHsfA7a*, *AcHsfA8b*, *AcHsfA9b*, and *AcHsfB2c*, exhibited higher expression levels during the post-pollination stage (**Figures 5B**, **S6**). On the contrary, genes like *AcHsfB1a*, *AcHsfB1c*, and *AcHsfB2a* showed higher expression levels during the post-harvest stage (**Figures 5B**, **S6**). To further understand the potential functions of *AcHsfs* in fruit development and response to ethylene treatment, we explored the expression profile under different fruit developmental stages with and without ethylene treatment (**Figure 5C**). Interestingly, the results displayed that the expression levels of multiple *AcHsfs*, including *AcHsfA1c*, *AcHsfA5a*, *AcHsfA5b*, *AcHsfA7a*, *AcHsfA8a*, *AcHsfA8b*, and *AcHsfA9b*, were down-regulated after ethylene treatment. Notably, ethylene treatment severely inhibited the expression of *AcHsfA9b*, while *AcHsfB1a* showed the opposite response (**Figure 5C**). This suggests that these *AcHsfs* might be involved in the regulation of fruit development and response to ethylene signaling. Additionally, the *AcHsfs* presented different expression profiles under low temperatures (**Figure S5**). *AcHsfA5b* and *AcHsfA9b* showed an increased expression with temperature increment. On the contrary, some *AcHsfs* (*AcHsfA3a*, *AcHsfB2c*, and *AcHsfC1b*) showed increased expression under low temperatures (**Figure S5**).

### 3.7 RT-qPCR validation of kiwifruit *Hsfs* under different temperature intervals

**Figure 6:**
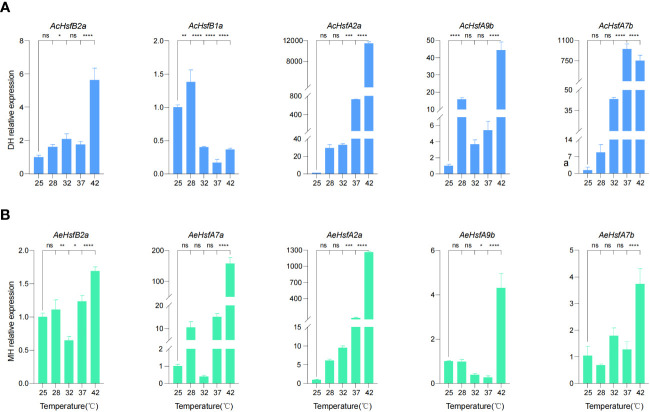
Expression profile of kiwifruit *Hsfs* under different intervals of temperature based on RT-qPCR results. **(A)** Relative expression of *AcHsfs*, and **(B)** Relative expression of *AeHsfs*. DH = ‘Donghong’, and MH = ‘Maohua no.1’. *AcActin* and *AeActin* were used as the internal standard for each gene. Data were shown as means ± SD (n=3). Significance was determined by one-way ANOVA with Dunnett posttest, and statistical significance is indicated by *(*p <*0.05), **(*p <*0.01), ***(*p <*0.001), ****(*p <*0.0001), ns (no significant difference).

We selected five *AcHsfs* and five *AeHsfs* from kiwifruit plants treated with different temperature intervals (25°C, 28°C, 35°C, 37°C, and 42°C) for 3 h for RT-qPCR analysis (**Figure 6**) (Wang et al., 2022). The plant samples used in this analysis were collected from the Ac cultivar ‘Donghong’ (DH) and the Ae cultivar ‘Maohua no.1’ (MH) (**Figure 6**). Our results illustrated that a temperature of 42°Csignificantly enhanced the expression levels of all selected candidate genes, except for *AcHsfB1a*, indicating that these genes are primarily responsive to high temperatures (**Figure 6**). Interestingly, the expression levels of two *AcHsf* genes (*AcHsfA2a* and *AcHsfA7b*) in DH and the *AeHsfA2a* in MH were significantly different at different temperature intervals (**Figure 6**). *AcHsfA2a* and *AeHsfA2a* displayed an upward trend with higher temperatures, whereas the expression levels of *AcHsfA7b* decreased after 37°C, suggesting that different kiwifruit cultivars exhibit distinct responses to varying temperatures (**Figure 6**).

### 3.8 Subcellular localization of kiwifruit Hsfs

**Figure 7:**
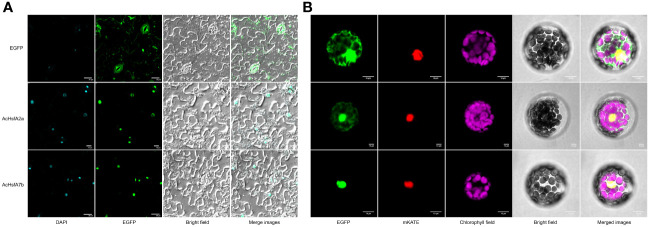
Subcellular localization of AcHsfA2a/AcHsfA7b. **(A)** Subcellular localization of the fusion protein 35S::AcHsfA2a/AcHsfA7b::EGFP in tobacco leaves, and **(B)** Subcellular localization of the fusion protein 35S::EGFP::AcHsfA2a/AcHsfA7b in mesophyll protoplasts of *Arabidopsis*.

Based on the differential expression patterns observed in RT-qPCR results, we selected two candidate genes, *AcHsfA2a* and *AcHsfA7b*, for sub-cellular localization analysis. *In-silico* subcellular localization analysis predicted their presence in the nucleus of plant cells. Experimental analysis in this study confirmed that both AcHsfA2a and AcHsfA7b were indeed localized in the nucleus of tobacco leaves and *Arabidopsis* protoplasts, regardless of whether the Hsf proteins were connected to the N-terminus or C-terminus of GFP (**Figure 7**). However, it is worth noting that a slight GFP-AcHsfA2a signal was also observed in the cytoplasm of *Arabidopsis* protoplasts (**Figure 7B**). In short, both GFP-AcHsfA2a/AcHsfA7b and AcHsfA2a/AcHsfA7b -GFP were primarily located in the nucleus.

### 3.9 High-temperature treatment significantly induced the expression of *AcHsfA2a*

**Figure 8:**
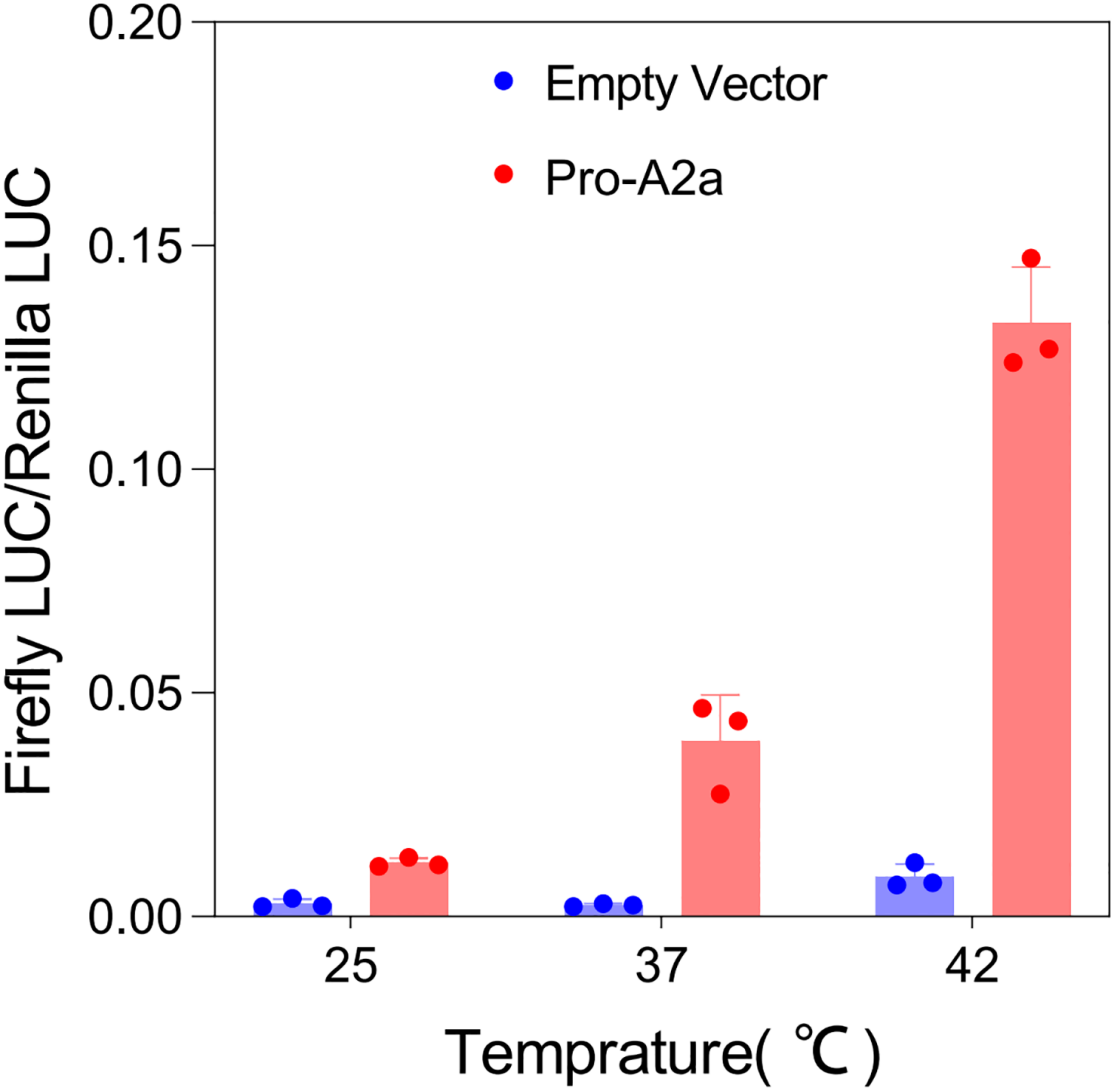
Dual-luciferase reporter assay of Pro-A2a under different intervals of temperature.

To further determine the response of *AcHsfA2a* to different temperatures, we conducted a transient expression assay using the pGreenII 0800-LUC-pro-AcA2a reporter vector in tobacco leaves. The results demonstrated a significant difference in the expression of *AcHsfA2a* compared to the control. As the temperature increased, there was a notable increase in the ratio of LUC/REN of pro-A2a -LUC (**Figure 8**), which suggests that heat stress has the ability to promote the transcription of *AcHsfA2a*.

## 4 Discussion

The anticipated increase in global warming in the near future warrants an in-depth understanding of the magnitude of heat stress damage in fruit plants (Jagadish et al., 2021). Throughout their lifecycle, plants encounter unfavorable environmental conditions, leading to the evolution of specific regulatory mechanisms to combat the devastating effects caused by these challenges (Tiwari et al., 2020). In recent years, plant responses to global warming have emerged as a research hotspot (Raza et al., 2019; Srivastav et al., 2021). High temperature, a global issue, disrupts the physiological and biochemical activities of plants, consequently reducing crop yields (Hasanuzzaman et al., 2012). The *Hsf* genes within plants regulate the expression of various stress-responsive genes to attain tolerance against a range of environmental factors, including heat stress (Ul Haq et al., 2019). So far, no identification has been reported of *Hsf* gene family members responsible for heat stress tolerance within kiwifruit species.

In the present study, we performed a genome-wide identification analysis of *Hsf* gene family members in two different diploid kiwifruit species (Ac and Ae), resulting in the identification of 36 and 41 *Hsf* genes within Ac and Ae genomes, respectively (**Table S1**). The discrepancies in the count of *Hsf* family members among the superfamilies and subfamilies of the two species suggested that they may have undergone distinct evolutionary patterns. The number of *Hsf* genes present within kiwifruit species exceeded the number identified in most other reported plants (**Table S3**), suggesting adequate preservation of these genes throughout the evolution process posing kiwifruit species. Interestingly, none of the identified kiwifruit *Hsf* genes clustered with HsfC2 (**Figure 2**). This suggests that during the evolution of the kiwifruit *Hsf* gene family, a loss of the homologous gene equivalent to HsfC2 occurred.

Furthermore, the conserved DBD comprised three α-helices (α1–3) and four β-sheets (β1–4) (Nover et al., 2001). The central portion of the DBD is made up of the helix-turn-helix motif (H2-T-H3) with a considerable number of amino acid residues invariant among different organisms (Wang et al., 2009). Motif 1 corresponded to highly conserved regions including the H2-T-H3 region (**Figure S4**). Adjacent to the DBD domain in the C-terminal, the HR-A/B region is characterized by a coiled-coil structure (coil-coil structure) (Nover et al., 2001). Motifs 4 and 5 corresponded to the coil-coil structure (**Figure S4**). Researchers have noted a remarkable diversity in the structure and function within the *Hsf* gene family in plants (Von Koskull-Doring et al., 2007). The structure richness of kiwifruit *Hsfs* played a significant part in their diverse functionality. Gene structure analysis demonstrated that all the kiwifruit *Hsfs* possessed similar motif composition, albeit with slight variances across groups—where some motifs were solely present within a specific subfamily of Group A. This observation underscores how kiwifruit *Hsf* genes have been well maintained throughout evolutionary processes. It suggests that kiwifruit *Hsfs* sharing similar conserved domains and motifs may perform similar functions related to heat stress tolerance. The occurrence of functional divergence among multigene family members throughout evolution is a commonly recognized phenomenon (Conant and Wolfe, 2008).

Leveraging transcriptome datasets, we discovered that *AcHsfs* exhibit highly tissue-specific expression patterns, which could directly influence their functions (Swindell et al., 2007). The response of plants to heat stress is particularly associated with regulation of phytohormones, which oversee various abiotic stresses (Zheng et al., 2023). Salicylic acid (SA), jasmonic acid (JA), ethylene, abscisic acid (ABA), and auxin (AUX) are reported to regulate the expression of *Hsfs* under abiotic stresses (Cao et al., 2023). The expression pattern of *AcHsfB1a* and *AcHsfA9b* was altered differently (**Figure 5C**), implying that *AcHsfB1a* might function as an inhibitor for *AcHsfA9b*. They may play crucial roles in regulating plant responses to abiotic stresses as well as contributing to growth and development.

Gene expression analysis bridges the inherent information encoded within a gene and its ultimate functional product (Segundo-Val and Sanz-Lozano, 2016). For instance, *A. thaliana* plants demonstrated an upregulated expression pattern for *AtHsfA2* and *AtHsfA7* at 37°C for 1 h (Busch et al., 2005). Unlike the *AthsfA2-1* mutants, the *AtHsfA7a* and *AtHsfA7b* mutants did not exhibit heat resistance deficiencies, and the defective phenotype of the *AthsfA2-1* mutants could not be restored by the *AtHsf7a* and *AtHsf7b* genes (Nover et al., 2001; Charng et al., 2007). We have quantified the expression of several genes in kiwifruit that are orthologous to those in *A. thaliana*. The relative expression profile has suggested that *AcHsfA2a* might play a more vital role than *AcHsfA7b* in heat stress tolerance in plants (**Figure 6**). Remarkably, the dramatic change in the expression of *AcHsfA2a* under different temperature conditions underscores the necessity to elucidate its function as a potential marker gene for heat stress tolerance (**Figures 6**, **8**). Moreover, the similar expression patterns of ortholog gene pair *AcHsfA2a* and *AeHsfA2a* suggested that they could function similarly in responding to high-temperature stress in plants (**Figure 6**).

In numerous instances, the C-terminal regions of HsfA displayed leucine-rich sequences that might function as NES (Kotak et al., 2004). Indeed, many Hsfs are known to shuttle between the nucleus and cytoplasm (Heerklotz et al., 2001). In this study, the AcHsfA2a-GFP was found positioned in the nucleus of tobacco leaves (**Figure 7A**). However, GFP-AcHsfA2a was located in both the nucleus and cytoplasm of *Arabidopsis* protoplasts (**Figure 7B**), aligning with the results observed with GFP-LlHsfA2b (Xin et al., 2017). This suggests that AcHsfA2a may play roles beyond transcriptional regulation. It’s plausible that the excessive expression of exogenous GFP-AcHsfA2a may not be entirely incorporated into the nucleus. Another possibility is that GFP-AcHsfA2a shares an equal propensity for localization in both the cytosol and the nucleus, suggesting that the GFP may mask the NES domain contained in the C-terminal of AcHsfA2a, thereby affecting its localization. We will be conducting a more detailed functional analysis of AcHsfA2a in the subsequent phase of our studies.

## 5 Conclusion

In this research, we executed a structural and functional characterization of *Hsf* family members within the genomes of both Ac and Ae. This allowed us to elucidate the phylogenetic relationships, conserved motifs, and conserved domains present in kiwifruit *Hsfs*. The expression profile of kiwifruit *Hsfs* indicated that their expression was highly tissue-specific. The WGD events played a notable role in the evolutionary trajectory of kiwifruit *Hsfs*. Subcellular localization analysis revealed that both AcHsfA2a and AcHsfA7b were positioned in the nucleus of the plant cell, with GFP-AcHsfA2a also detectable within the cytoplasm in *Arabidopsis* protoplasts. Further, RT-qPCR analysis demonstrated that most *Hsf* genes were responsive to high-temperature conditions. Dual-Luciferase assay results indicated an upregulation in the expression of *AcHsfA2a* under heat stress. Collectively, these findings lay a theoretical foundation for the functional validation of candidate genes related to heat stress tolerance in kiwifruit and other plant species, offering valuable insights for future endeavors in breeding heat-tolerant kiwifruit cultivars.

## Data availability statement

The datasets presented in this study can be found in online repositories. The names of the repository/repositories and accession number(s) can be found in the article/**Supplementary Material**.

## Author contributions

ZW and PG conceived the research. JL, YZ, EY, and XC performed the experiments and analyzed the data; JT, MA, and ZW wrote the manuscript. ZW and HH initiated the study ideas and revised the manuscript. All authors contributed to the article and approved the submitted version.
