# Supplementary figures and images for: Genome-wide identification of the heat shock transcription factor gene family in two kiwifruit species
Source: Front Plant Sci. 2023 Sep 20;14:1075013. doi: 10.3389/fpls.2023.1075013 (PMC10548268; doi:10.3389/fpls.2023.1075013)

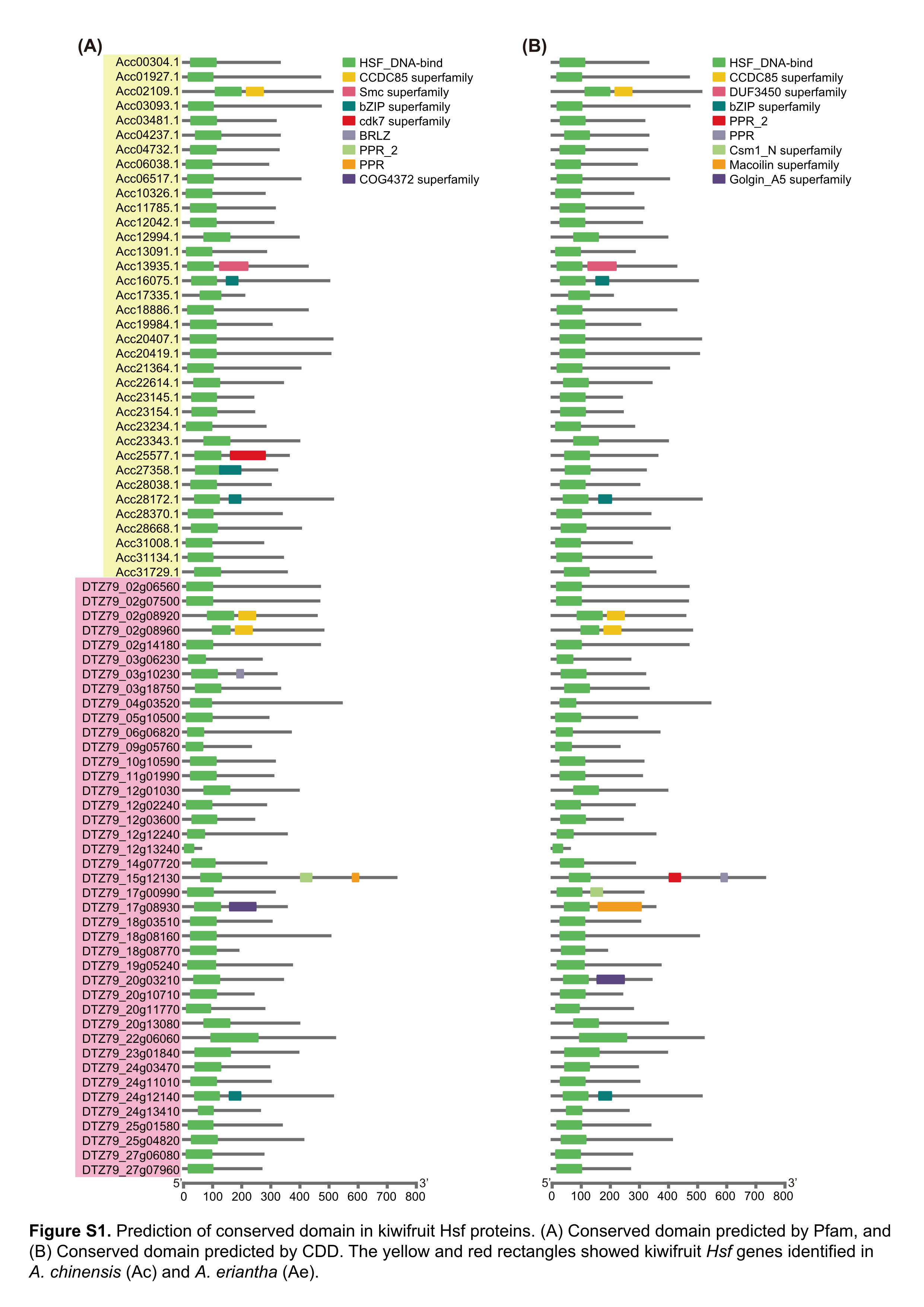

Supplement: Supplementary file 1 [file Image_1.tif]

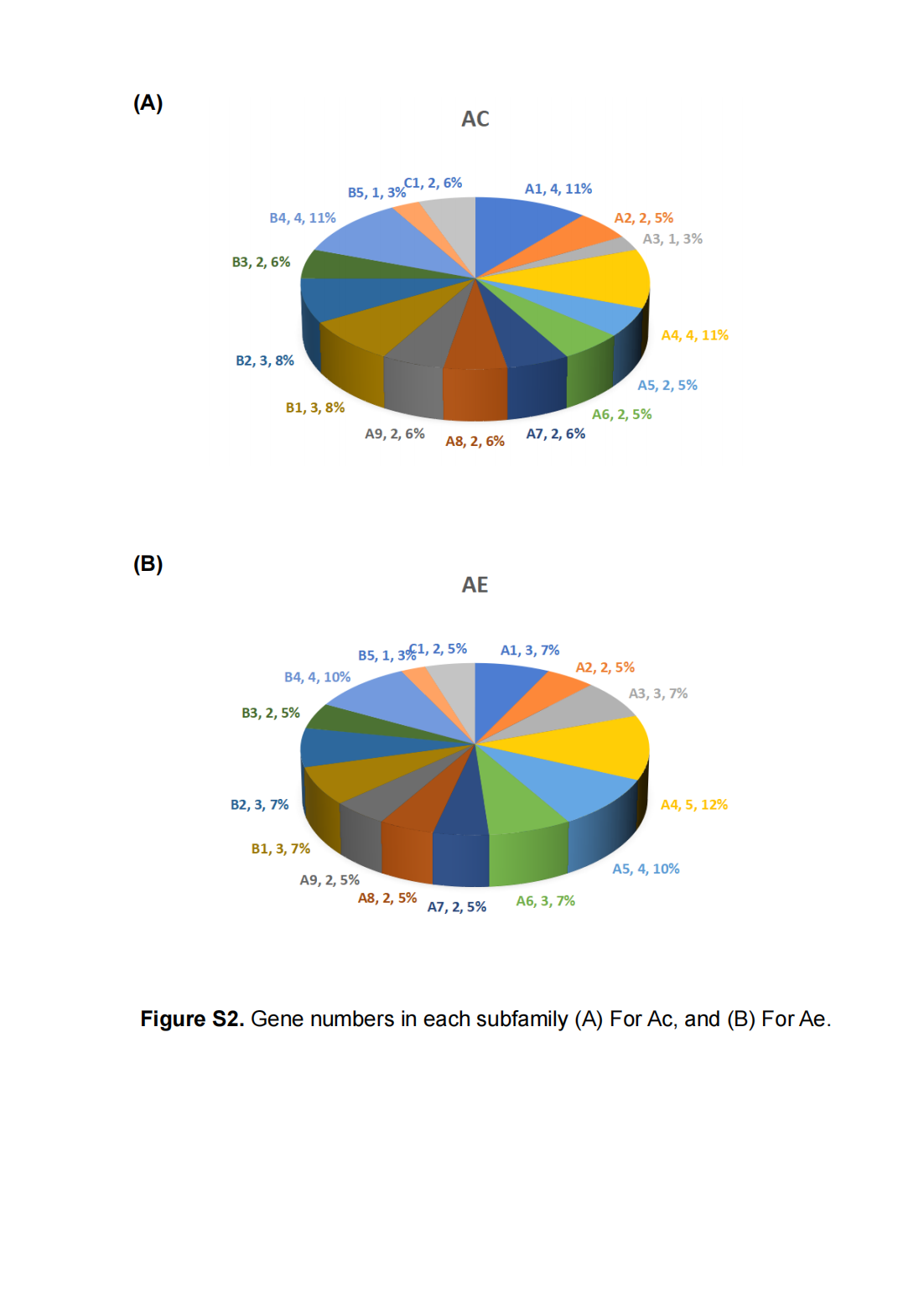

Supplement: Supplementary file 2 [file Image_2.tif]

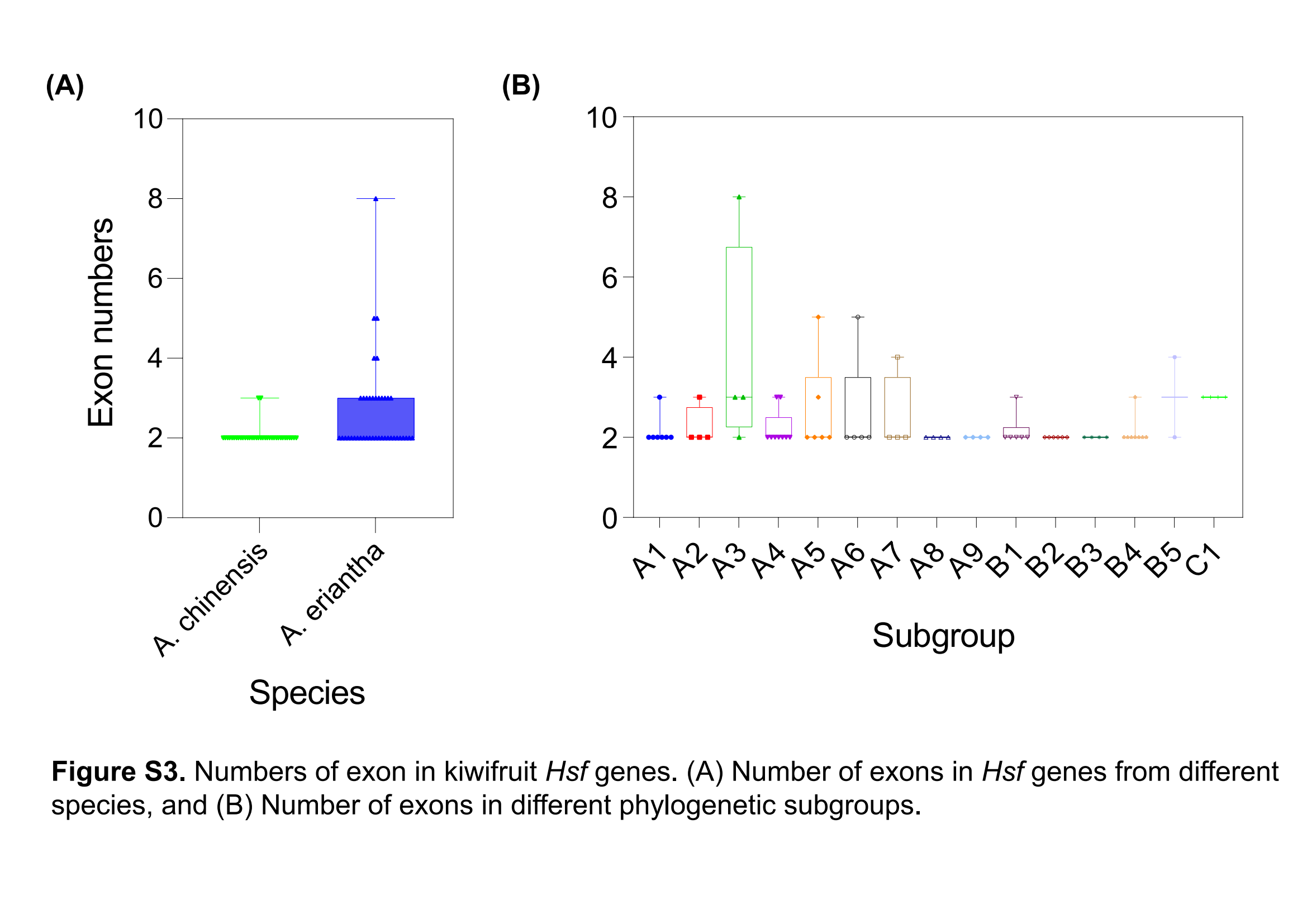

Supplement: Supplementary file 3 [file Image_3.tif]

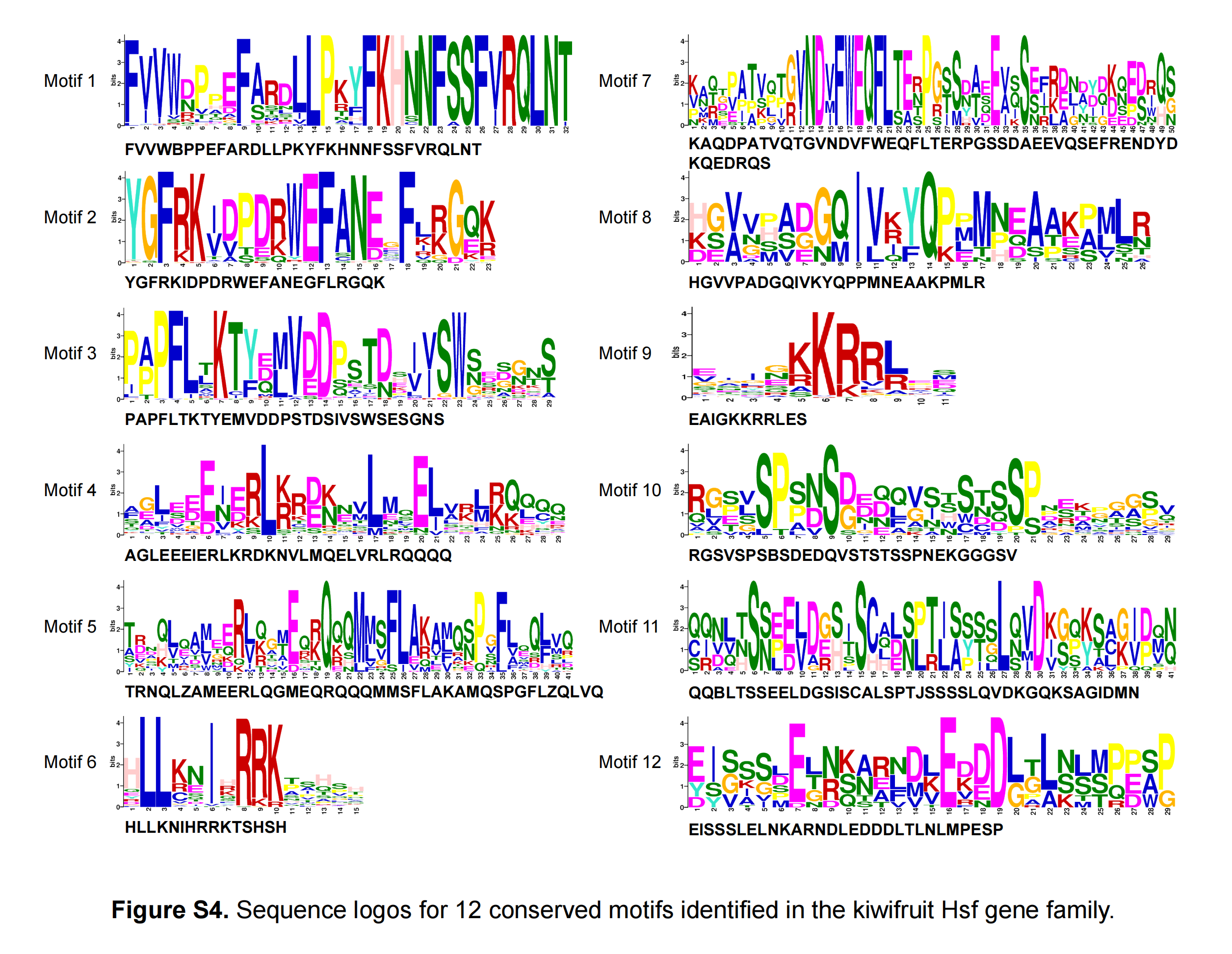

Supplement: Supplementary file 4 [file Image_4.tif]

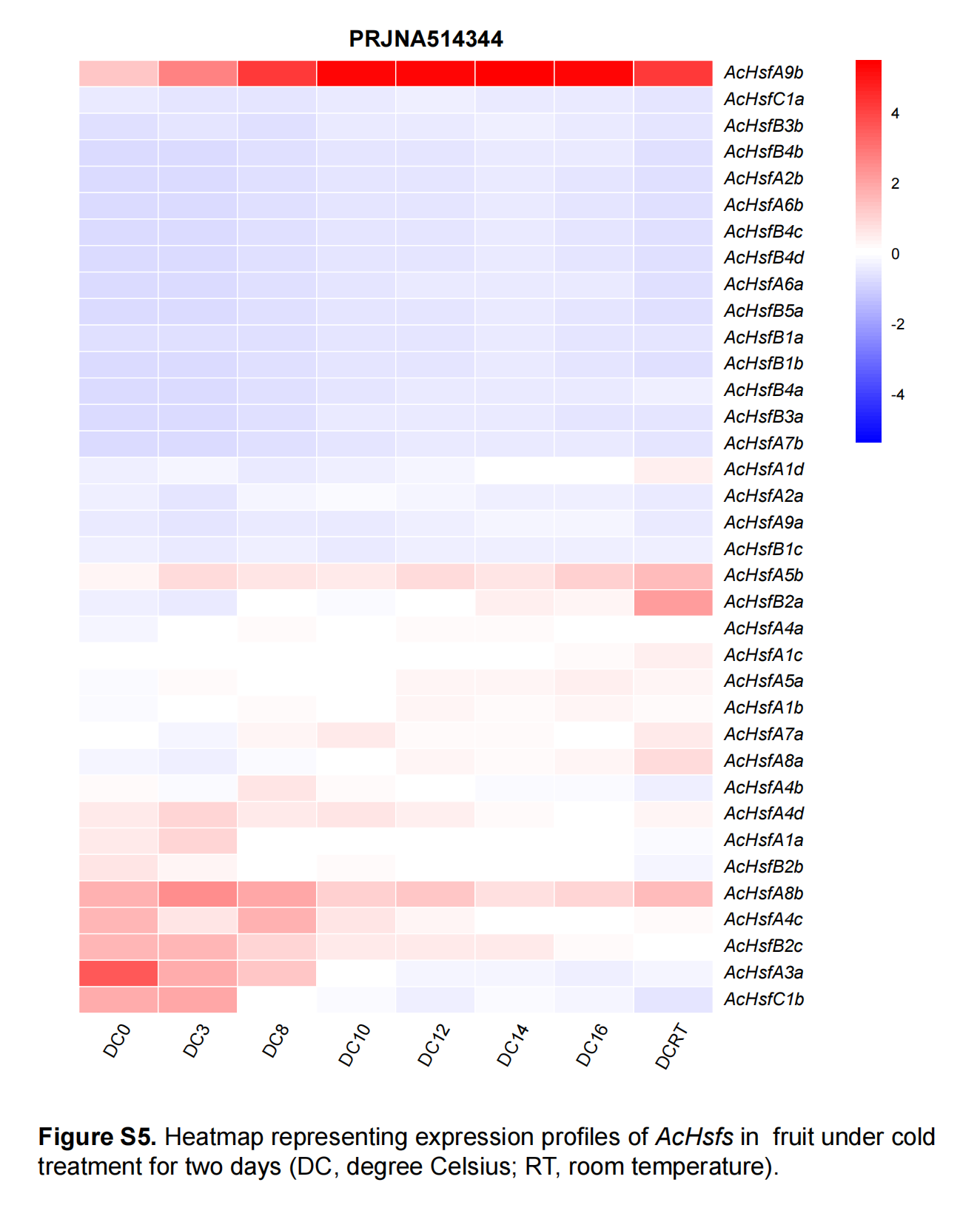

Supplement: Supplementary file 5 [file Image_5.tif]

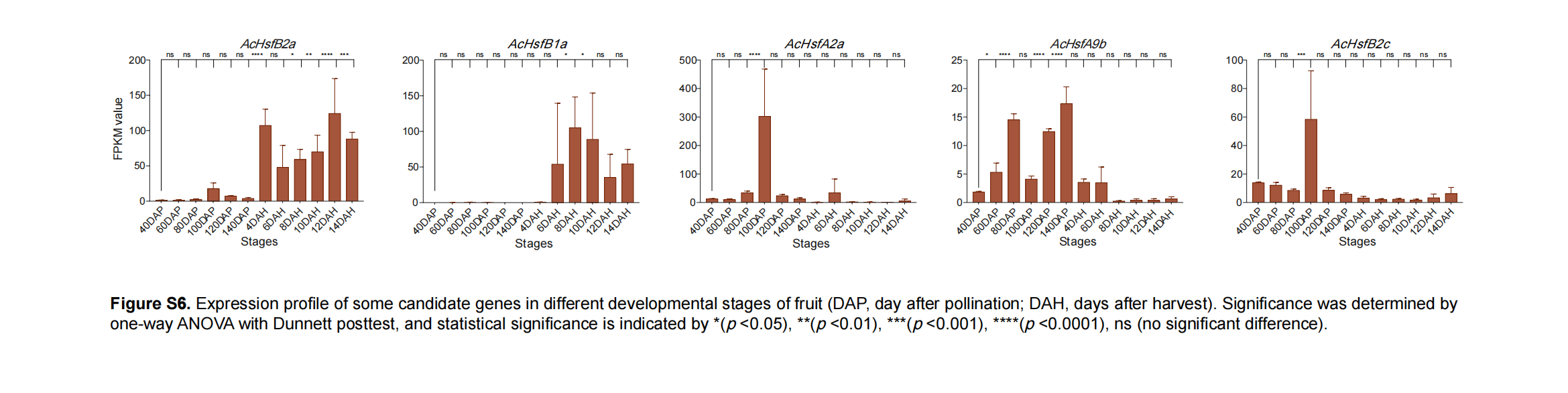

Supplement: Supplementary file 6 [file Image_6.tif]
